# Novel SNPs in the leptin gene: implications for growth performance in cultured European sea bass *(Dicentrarchus labrax)*

**DOI:** 10.2478/jvetres-2026-0021

**Published:** 2026-04-18

**Authors:** Emel Özcan Gökçek

**Affiliations:** Department of Aquaculture, Faculty of Fisheries, Ege University, 35100 İzmir, Türkiye

**Keywords:** candidate gene, growth trait, haplotype, *lep* gene, teleost fish

## Abstract

**Introduction:**

Leptin, a class I helical cytokine, regulates growth, appetite, energy homeostasis and reproductive functions in fish. The leptin gene (*lep*) is a candidate gene for growth trait influence in both farm animals and teleost fish.

**Material and Methods:**

This study investigated variations in the *lep* gene and their associations with growth-related traits in European sea bass (*Dicentrarchus labrax*) using DNA sequencing.

**Results:**

Twelve novel single-nucleotide polymorphisms (SNPs) were identified in the first and second introns and the third exon of the gene, including a non-synonymous third exon variant (g.11004767C>T) causing an arginine-to-tryptophan substitution. This locus was significantly associated with multiple growth traits, including total and fillet weights and standard, body and post-anal lengths (P-value < 0.05). Analysis revealed two haploblocks; block 1 having four haplotypes (GCG, GTG, ACA and GTA), with GTA associated with the highest total and fillet weights and body length measurements (P-value < 0.01). Block 2 comprised three haplotypes (AG, GG and AA), where AG carriers exhibited superior growth to GG and AA carriers (P-value < 0.01). *In silico* analysis of the non-synonymous third exon variant (g.11004767C>T) suggested its potential effects on local protein conformation and receptor binding.

**Conclusion:**

These findings indicate that *lep* gene polymorphisms, particularly the g.11004714G>A locus and specific haplotypes, are associated with growth variability in European sea bass. The identified SNPs and haplotypes may facilitate molecular marker–assisted selection in growth performance breeding programmes in economically important aquaculture species.

## Introduction

The European seabass (*Dicentrarchus labrax*) belongs to the Moronidae family and Perciformes order. It is a coastal marine species distributed throughout the north-eastern Atlantic Ocean, the Mediterranean Sea and the Black Sea. The European seabass is among the most important cultured species in Mediterranean and European aquaculture, where it is extensively farmed and in high demand for its economic and nutritional value (36). It typically reaches market size within approximately 24 months and then reaches consumers who value it highly for its superior flesh quality and take up a total aquaculture production which reached 286,917.69 tonnes in 2023 (11). The species’ attractiveness in aquaculture is the encouragement to improve its productivity and yield. These are directly affected by growth traits; therefore, selecting for the best growth traits is essential in aquaculture. As a complex quantitative trait, growth is regulated by multiple genes and influenced by multifactorial molecular networks (8).

Leptin is a class I helical cytokine hormone, known as an anti-obesity hormone that regulates energy balance, body weight, reproduction and immune function through hypothalamic pathways in vertebrates, teleost fish included (21, 40). In fish it is expressed in adipose tissue, the ovaries, the pituitary gland, and predominantly in the liver (21). The tissue-specific expression pattern of leptin indicates functional diversity among organs and species, reflecting the greater metabolic flexibility of teleosts compared to mammals and suggesting a broader regulatory role for leptin in fish physiology (12). In *D. labrax*, plasma leptin levels correlate with body-fat reserves and fluctuate under different nutritional conditions, as evidence of leptin’s role in metabolic regulation, growth and energy homeostasis associated with reproductive function in this species (10). A genome-wide association study identified a significant regulatory role for the leptin gene in feed intake and energy balance in olive flounder (*Paralichthys olivaceus*) in a temperature-tolerance study (35). Previous genomic investigations have suggested that variations within the *lep* region may contribute to growth diversity among individuals. In European sea bass, linkage group 6, which contains the *lep* gene, has been reported to significantly influence growth performance (20). In turbot (*Scophthalmus maximus*), a genetic marker located on LG16 accounted for up to 12.9% of the phenotypic variance in growth and was found to be closely associated with *lep* and other growth-related genes (31). Furthermore, 34 *lep*-associated SNPs have been identified across multiple linkage groups in growth hormone–transgenic coho salmon (*Oncorhynchus kisutch*), indicating the extensive genomic influence of *lep* on growth regulation (22). Collectively, these results demonstrate that *lep* polymorphisms play a role in growth variation and support the gene’s potential use as a candidate marker for selective breeding programmes.

Sustainable development of the aquaculture industry necessitates the implementation of genetic selection programmes, which require a comprehensive understanding of cultured species’ genomic background. In marker-assisted selection programmes, single-nucleotide polymorphisms (SNPs) within candidate gene regions are identified using molecular techniques, and their potential associations with economically important traits are evaluated (34). Because SNPs are highly stable biallelic and occasionally triallelic markers that can occur in both coding and non-coding regions of the genome, they are particularly useful for genetic studies (30). They have become valuable molecular markers in aquaculture genetics, facilitating improvements in growth performance, disease resistance and other economically important traits, and thereby driving the sustainable development of aquaculture (25, 26, 37). These polymorphisms can also generate haplotypes associated with phenotypic variation, particularly in growth-related characteristics (14).

Among the various candidate genes, *lep* has attracted considerable attention because of its multifunctional role in regulating energy balance, metabolism and growth. In terrestrial livestock, particularly in cattle and buffalo, *lep* polymorphisms have been extensively studied and are known to influence carcass composition, body weight regulation, feed efficiency, milk yield and immune function (16). Studies on *lep* gene polymorphisms are now becoming increasingly common in aquaculture species as well. For instance, Tang *et al*. (33) demonstrated that polymorphisms in this gene are associated with growth traits and can be applied in marker-assisted selection programmes in carp (*Cyprinus carpio* var. Jian). Similarly, Sciara *et al*. (32) identified *lep* and *fgf6* as key growth-related genes in turbot through genome mining. In this context, the *lep* gene may represent a promising candidate for growth regulation and metabolic control in *D. labrax*. To the best of my knowledge, this study represents the first investigation of *lep* gene polymorphisms and their association with growth-related traits in European sea bass, addressing a major knowledge gap in aquaculture genetics compared to terrestrial livestock species.

Beyond the *lep* gene’s metabolic and regulatory functions, influence upon growth-related traits may also be one of its activities, as indicated by accumulating genomic evidence attributing this to polymorphisms in the *lep* region. However, such polymorphisms and growth-related traits have not yet been reported in *D. labrax*. The aims of this study were to identify polymorphisms in the *lep* gene and evaluate the relationship between genotypes and growth traits, acknowledging the gene’s importance in regulating metabolism and growth in European sea bass. In addition, the potential effects of SNPs located in the coding region of the *lep* gene on the three-dimensional structure of leptin protein were analysed.

## Material and Methods

### Sample collection and DNA extraction

The European sea bass used in this study were obtained from a broodstock tank containing approximately 30 females and 30 males which spawned on the same day. After reaching an average body weight of 2.5 g within four months, the fish were transferred to an offshore sea cage located in Çeşme, İzmir. During the 20-month grow-out period in the sea cages, the fish were fed a commercial diet containing 35–45% protein and 20–30% lipid until the end of the rearing period. At harvest, the fish were transported to a fish processing facility, where 400 same-aged *Dicentrarchus labrax* individuals were randomly sampled. Sex was determined at the time of sampling, and all individuals were identified as males. *D. labrax* is a gonochoristic species with a polygenic sex determination system influenced by environmental factors; under aquaculture conditions, exposure to certain temperature regimes during early development often increases the proportion of males, resulting in 75–100 % male-biased populations (10, 36). The effects of sex were ignored in the analysis. Fish samples were transported to the Breeding and Molecular Genetics Laboratory of the Department of Aquaculture of Ege University in Izmir, where they were photographed and subsequently measured for total body and fillet weight using an electronic balance with precision of 0.01 g. The Digimizer tool was used to measure the ground truth distances in the original image (MedCalc, Ostend, Belgium). Morphometric analyses were conducted, and the following measurements were recorded for each fish: total weight, standard length, body length, head length, pectoral–anal length, abdominal length, post-anal length, head height and body height. After dissection, the muscle tissue from each fish was stored at –20°C until DNA extraction. A High Pure PCR Template Kit (Roche, Mannheim, Germany) was used to extract genomic DNA from frozen muscle tissue, and the quantity of the extracted total DNA was estimated using a spectrophotometer.

Since the sampling was performed *post* harvest, ethical approval was not required at this stage according to the guidelines of the Ege University Animal Ethics Committee.

### Primer design and PCR amplification

Primer sequences of the *lep* gene to permit investigation of all regions including all exons and introns were designed based on the European sea bass sequence retrieved from GenBank (accession No. CAJNNU010000020) using the Primer-BLAST (basic local alignment search tool) algorithm (Table 1). A PCR was performed in a 50 μL reaction volume containing 50 ng of genomic DNA, 0.5 μM of each primer, 2× MyTaq™ Mix (5 μL) (Meridian Bioscience, Cincinnati, OH, USA), and 1 U (0.25 μL) of Taq Hot Start DNA polymerase (Bioline, part of Meridian Bioscience, Memphis, TN, USA). The thermal cycling protocol consisted of an initial denaturation step at 95°C for 4 min followed by 35 amplification cycles. Each cycle included denaturation at 95°C for 40 s, annealing at 59°C and 61°C for 45 s, and an extension at 72°C for 90 s (Table 1). Finally, a 10-min extension step at 72°C was carried out to ensure complete amplification. The PCR products were analysed on a 1.5% agarose gel by horizontal electrophoresis, and the gels were stained using RedSafe (iNtRON, Seongnam, South Korea) (Table 1).

**Table 1.** Primers used to amplify 1,655 base pairs of the *Dicentrarchus labrax lep* gene sequence

Gene region	Primer sequence (5′–3′)	Annealing	Sequenced regions of *lep* gene of *Dicentrarchus labrax*	Length
*Lep*-1	F: GTTGGTTAGAGGGAGCTGT R: ACCATTGTCTCAGCTCACCA	59°C	Exon 1 and partial region of intron 1	849 bp
*Lep-*2	F: CAGGGGGAGAATGCAAAGT R: CATTCAGTTCAGTTCCCCGT	61°C	Partial region of intron 1, exon 2, intron 2 and exon 3	856 bp

### Sequencing, SNP identification and genotyping

The *D. labrax lep* gene was sequenced using a 3500XL Genetic Analyzer System (Applied Biosystems, Foster City, CA, USA). The sequences were checked using ChromasPro v. 2.1.10.1 (Technelysium, South Brisbane, QLD, Australia). Sequence trimming and alignment were performed using BioEdit v. 7.2.5 (13). The *lep* gene fragments were validated using BLAST at the NCBI website.

Population genetic parameters for the 400 individuals were expected heterozygosity, observed heterozygosity, minor allele frequency (MAF) and polymorphic information content (PIC). Hardy–Weinberg equilibrium (HWE) calculations were performed using a custom web-based tool following the GenAlEx methodology (27). Linkage disequilibrium (LD) and haplotype analyses of loci with SNPs were performed using Haploview v.4.2 software (3).

### Association analysis

The associations between genotypes, haplotypes and growth traits were analysed using a general linear model (GLM) and SPSS software v. 25.0 (IBM, Armonk, NY, USA). The linear model was:

$$I=Y_{jk}=\text{μ}+G_{j}+e_{jk}$$

where *Y_jk_* represents the trait being analysed; μ denotes the overall mean, *G_j_* corresponds to the fixed effect of the *lep* genotype or haplotype (*j* = 1, 2 or 3 for each SNP or haplotype) and *e_jk_* represents the random error term.

The Bonferroni multiple range test was used to assess the significance of the differences between genotypes at each locus. The significance threshold was set at P-value < 0.05 for statistically significant differences and P-value < 0.01 for highly significant differences. Genotypes and haplotypes with a frequency of less than 0.03 were excluded from the association analysis. Correction for multiple testing of SNPs associated with growth traits was performed using the Benjamini–Hochberg false discovery rate approach (4).

### *In-silico* translation and structural modelling of the *lep* gene

The nucleotide sequence of the *lep* gene region was translated using Expasy Resource Portal (9). The three-dimensional structure of the *Dicentrarchus labrax* leptin isoform X1 protein was predicted using the ColabFold tool implementing the AlphaFold algorithm (18) and visualised using PyMOL v.3.0.5. (Schrödinger, New York, NY, USA). Using the PyMOL mutagenesis wizard, the native arginine at position 159 was replaced with tryptophan. Conserved residues were identified using ConSurf (41). Structural interactions and bond lengths in the vicinity of residue 159 were analysed. The electrostatic potential distributions of native and mutant proteins were calculated and compared using the Adaptive Poisson–Boltzmann Solver (2). Analysis in the I-Mutant2.0 database (6) was performed using the amino acid sequence of the protein along with the position of the substituted residue. For Missense3D (17) analysis, the three-dimensional structure of the leptin isoform X1 protein was uploaded to the database.

## Results

### Summary of growth traits

The descriptive statistics presented in Table 2 indicate substantial variation in the growth performance of the European sea bass population studied. Total weight (171.05–863.60 g) and fillet weight (81.11–417.44 g) exhibited relatively high coefficients of variation (CV = 34%), reflecting considerable individual differences in growth potential. In contrast, body length and standard length displayed much lower variability (CV = 12%), suggesting more uniform body proportions compared to weight-related traits. The mean total weight (459.35 ± 156.97 g) and mean body length (21.54 ± 2.55 cm) indicate overall satisfactory growth performance under the applied culture conditions. Collectively, the observed range and variability of the production traits suggest adequate phenotypic diversity within the sampled population, providing a robust basis for subsequent genetic association and genotype–phenotype analyses.

**Table 2.** Descriptive statistics for production traits

Traits	Min	Max	Mean (SD)	CV (%)
TW (g)	171.05	863.60	459.35 (156.97)	34
FW (g)	81.11	417.44	243.68 (82.99)	34
SL (cm)	21.38	36.47	29.41 (3.46)	12
BL (cm)	15.45	27.47	21.54 (2.55)	12
HL (cm)	5.21	18.87	8.10 (1.56)	19
PecAL (cm)	10.94	26.91	21.19 (2.92)	14
AL (cm)	7.67	17.73	13.59 (1.91)	14
PostAL (cm)	4.55	11.63	9.13 (1.22)	13
HH (cm)	3.65	6.88	5.05 (0.72)	14
BH (cm)	5.17	10.02	7.45 (1.04)	14

1 CV – coefficient of variation. TW –total weight; FW – fillet weight; SL –standard length; BL – body length; HL – head length; PecAL – pectoral–anal length; AL – abdominal length; PostAL – post-anal length; HH – head height; BH – body height

### Polymorphisms of *lep*

**Fig. 1.**
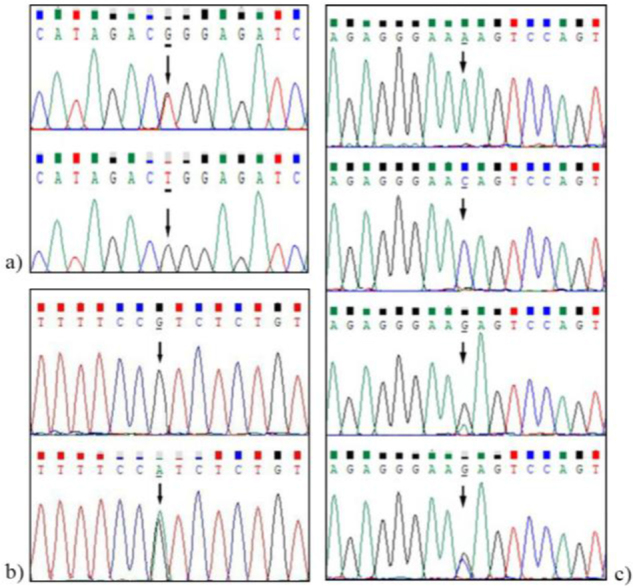
Partial sequence of polymorphisms in the *lep* gene of *Dicentrarchus labrax*. (a) g.11005646A>C; (b) g.11004767C>T; (c) g.11005020T>C>G. Chromatograms were generated from the reverse sequence

Citing details from the GenBank record under accession No. NW_026136720, the structure of the *lep* gene in *D. labrax* consists of three exons and two introns, and encodes a total of 191 amino acids. In the present study, a total of 1,655 base pairs (bp) of the *lep* gene were amplified, encompassing the first, second and third exons, and the first and second introns. Twelve novel SNPs were identified (Table 3 and Fig. 1), these being nine SNPs in the first and second introns and three SNPs located in the third exon. The g.11004804G>A and g.11004714G>A transition mutations are synonymous transitions, resulting in no changes to the encoded amino acids, glutamic acid and lysine respectively. The 11004767C>T mutation is a non-synonymous transition that causes an amino acid substitution from arginine (R) to tryptophan (W) (Fig. 1b). A tri-allelic SNP (g.11005020T>C>G) was identified in the second intron of the *D. labrax lep* gene (Fig. 1c), with the following genotypes: TC (n = 160), CG (n = 108), TG (n = 68), CC (n = 32), TT (n = 20) and GG (n = 16). However, this variant was excluded from the downstream analyses because the genotyping and statistical analyses used in this study were optimised for biallelic SNPs (5).

**Table 3.** Single-nucleotide polymorphisms (SNPs) identified in the *Dicentrarchus labrax lep* gene

SNP	Position	Ref. seq.	SNP site	Region	Mutations
1	11005825*	T	C>T>Y	Intron 1	-
2	11005698*	C	T>C>Y	Intron 1	-
3	11005646*	A	C>A>M	Intron 1	-
4	11005643*	C	T>C>Y	Intron 1	-
5	11005525*	C	A>C>M	Intron 1	-
6	11005514*	C	T>C>Y	Intron 1	-
7	11005335*	G	A>G>R	Intron 1	-
8	11005304*	A	G>A>R	Intron 1	-
9	11005020*	T	C>G>T>B	Intron2	-
10	11004804*	G	A>G>R	Exon 3	Synonym (glutamic acid)
11	11004767*	C	T>C>Y	Exon 3	Non synonym (arginine → tryptophan)
12	11004714*	G	A>G>R	Exon 3	Synonym (lysine)

* – position numbered according to GenBank reference sequence NW_026136720 (minus strand)

Polymorphism parameters of the five SNP loci were assessed to determine their genetic variability. The estimated PIC values ranged from 0.238–0.350 (Table 4). Four of the five SNPs exhibited moderate genetic diversity (0.25 < PIC < 0.50), suggesting a reasonable potential for application in breeding selection programmes, whereas one SNP showed low diversity (PIC < 0.25), and no highly polymorphic loci were detected in it. The MAF of these SNPs varied between 0.185 and 0.345, indicating that both alleles were present at appreciable frequencies. All analysed SNPs deviated significantly from HWE (P-value < 0.05). Six SNPs located between positions g.10058525T>C and g.11005514C>T in the first intron were excluded from the analyses because of their very low frequency in the studied population. For the same reason, genotypes and haplotypes with a frequency lower than 0.03 were excluded from the association analysis to ensure sufficient statistical power (34). Although twelve SNPs were initially detected, only five loci were retained for further analysis. Six SNPs were excluded due to very low allele frequencies and one SNP showed a triallelic pattern. The remaining five SNPs were located in two haplotype blocks, consisting of three positions in block 1 and two positions in block 2.

**Table 4.** Genetic diversity parameters based on single-nucleotide polymorphisms in the *Dicentrarchus labrax lep* gene

Locus	Number of samples	MAF	H_e_	H_o_	PIC	HWE
11004714G>A	GG: 252 GA: 140 AA: 8	0.195	0.314	0.350	0.265	0.021*
11004767C>T	CC: 124 CT: 276	0.345	0.452	0.690	0.350	0.000*
11004804G>A	GG: 200 GA: 192 AA: 8	0.260	0.385	0.480	0.311	0.000*
11005304A>G	AA: 208 AG: 192	0.240	0.365	0.480	0.298	0.000*
11005335G>A	GG: 292 GA: 84 AA: 24	0.165	0.276	0.210	0.238	0.000*

1 MAF – minor allele frequency; H_e_ – expected heterozygosity; H_o_ – observed heterozygosity; PIC – polymorphic information content; HWE – Hardy-Weinberg equilibrium;

* –statistically significant differences (P-value < 0.05)

**Fig. 2.**
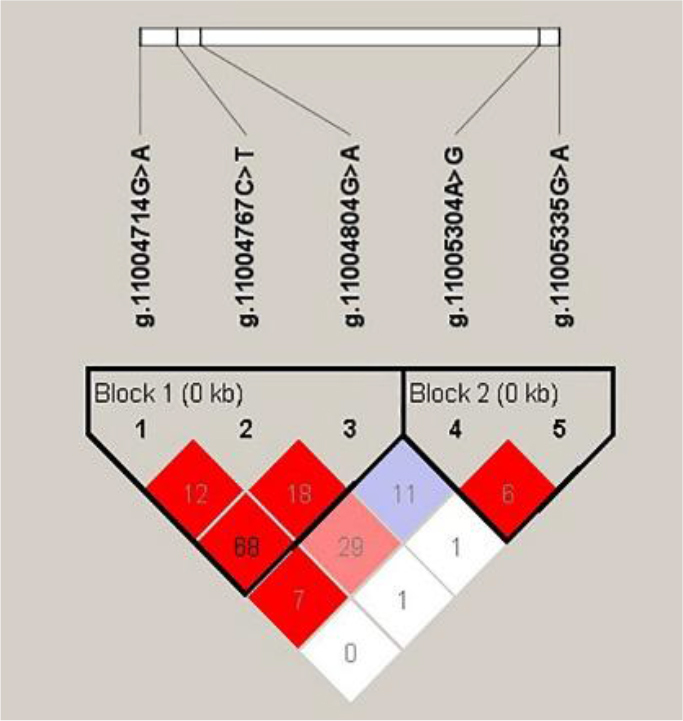
Linkage disequilibrium plot of the *Dicentrarchus labrax lep* gene. The values represent r^2^ × 100

Haplotype analysis identified two haplotype blocks based on the five significant SNPs of the *lep* gene, and their associations with growth traits were evaluated. Block 1 (positions g.11004714, g.11004767 and g.11004804) comprised six haplotypes (GCG, GTG, ACA, GCA GTA and ATA), whereas block 2 (positions g.11005335 and g.11005304) consisted of three haplotypes (AG, GG and AA). The LD plot of these five SNPs further confirmed the presence of two haplotype blocks with values corresponding to r^2^ × 100. Moderate LD was observed between g.11004714G>A and g.11004767C>T (r^2^ = 0.68) (Fig. 2). Haplotype analysis revealed that GCG and AG were the most frequent haplotypes in blocks 1 and 2, respectively, suggesting their potential predominance in the studied population.

### Association analysis between SNPs and growth-related traits

Significant associations between *lep* genotypes and certain morphological traits were identified. The five traits in the GA genotype g.11004714G>A were associated with significantly greater specific growth than those in the GG and AA genotypes (P-value < 0.05). The genotypes at the g.11004714G>A locus were found to be significantly associated with total weight (513.35 ± 25.37 g), fillet weight (273.60 ± 13.39 g), standard length (30.44 ± 0.55 cm), body length (22.40 ± 0.40 cm) and post-anal length (9.54 ± 0.19 cm) (P-value < 0.05) (Table 5). Additionally, genotypes at locus g.11004804G>A were associated with post-anal length (9.50 ± 0.16 cm) (P-value < 0.05). No significant associations were detected between *lep* genotypes and head length, pectoral–anal length, body depth, abdominal length or head height.

**Table 5.** Associations between the *lep* genotypes and the growth traits of *Dicentrarchus labrax*

Trait	Locus	Genotype			P-value
		GG	GA	AA	
TW (g)	g.11004714 G>A	433.72 ±19.60^ab^	513.35 ±25.37^a^	359.98 ±19.11^b^	0.033
FW (g)	g.11004714 G>A	229.57 ± 10.34^ab^	273.60 ±13.39^a^	187.70 ±7.58^b^	0.024
SL (cm)	g.11004714 G>A	28.67 ±0.43^ab^	30.44 ± 0.55^a^	26.80 ±2.40^b^	0.027
BL (cm)	g.11004714 G>A	21.10 ±0.34^ab^	22.40 ±0.40^a^	19.34 ±1.75^b^	0.022
PostAL (cm)	g.11004714 G>A	8.95 ±0.15^ab^	9.54 ±0.19^a^	8.23 ±0.81^b^	0.027
	g.11004804 G>A	8.84 ±0.16^ab^	9.50 ±0.16^a^	8.22 ±0.80^b^	0.011

1 TW – total weight; FW – fillet weight; SL – standard length; BL – body length; PostAL – post-anal length;

a,b –different superscripts within the same row indicate significant difference at P-value < 0.05

Two haploblocks were identified within the analysed SNP regions. In block 1, six haplotypes were initially detected; however, two haplotypes with frequencies below 0.03 were excluded from further analysis. The remaining four haplotypes (GCG, GTG, ACA and GTA) were correlated with significant differences in growth performance (P-value < 0.01) (Table 5). Fish carrying the GTA haplotype achieved the highest total (554.56 ± 34.78 g) and fillet weights (286.63 ± 19.12 g) and greatest body lengths. Individuals with the GCG and GTG haplotypes grew relatively less. Three haplotypes (AG, GG and AA) were identified in Block 2. Fish with the AG haplotype demonstrated consistently superior growth performance across all measured traits, including total and fillet weights and morphometric parameters, compared to those carrying the GG and AA haplotypes (P-value < 0.01) (Tables 6 and 7).

**Table 6.** Characterisation of haplotypes and association analysis with weight and primary body growth traits in *Dicentrarchus labrax*

	Haplotype	Frequency	TW (g)	FW (g)	SL (cm)	BL (cm)	BH (cm)
Block 1	GCG	0.453	456.30 ± 11.06^b^	244.20 ± 5.97^b^	29.03 ± 0.23^b^	21.54 ± 0.18^b^	7.50 ± 0.07
	GTG	0.287	435.37 ± 17.78^b^	227.18 ± 9.06^ab^	29.35 ± 0.39^b^	21.16 ± 0.28^b^	7.28 ± 0.12
	ACA	0.177	494.58 ± 12.79^ab^	265.17 ± 7.04^ab^	30.18 ± 0.28^ab^	22.10 ± 0.22^ab^	7.57 ± 0.09
	GTA	0.040	554.56 ± 34.78^a^	286.63 ± 19.12^a^	31.27 ± 0.86^a^	22.98 ± 0.58^a^	7.79 ± 0.18
Block 2	AG	0.595	500.73 ± 10.47^a^	265.28 ± 5.37^a^	30.14 ± 0.23^a^	22.09 ± 0.17^a^	7.75 ± 0.07^a^
	GG	0.240	402.60 ± 16.48^b^	215.41 ± 6.32^b^	28.74 ± 0.28^b^	21.01 ± 0.19^b^	7.13 ± 0.09^b^
	AA	0.165	392.69 ± 18.15^b^	206.90 ± 10.56^b^	27.73 ± 0.41^b^	20.32 ± 0.29^b^	6.82 ± 0.10^b^

1 TW – total weight; FW – fillet weight; SL – standard length; BL – body length; BH – body height;

a,b –different superscripts within the same row indicate significant difference between haplotype blocks at P-value < 0.01

**Table 7.** Characterisation of haplotypes and association analysis with segmental body growth traits in *Dicentrarchus labrax*

	Haplotype	Frequency	HL (cm)	PecAL (cm)	AL (cm)	PostAL (cm)	HH (cm)
Block 1	GCG	0.453	8.25 ± 0.13	21.05 ± 0.21	13.52 ± 0.15b	9.12 ± 0.10^b^	5.04 ± 0.04
	GTG	0.287	7.92 ± 0.14	21.06 ± 0.32	13.44 ± 0.19b	8.93 ± 0.12^ab^	5.01 ± 0.09
	ACA	0.177	8.17 ± 0.01	21.75 ± 0.24	13.95 ± 0.17^ab^	9.47 ± 0.10^ab^	5.22 ± 0.06
	GTA	0.040	8.33 ± 0.34	22.75 ± 0.71	14.66 ± 0.42^a^	9.66 ± 0.32^a^	5.26 ± 0.14
Block 2	AG	0.595	8.29 ± 0.11^a^	21.70 ± 0.20^a^	13.93 ± 0.13^a^	9.37 ± 0.08^a^	5.24 ± 0.05^a^
	GG	0.240	8.03 ± 0.13^a^	20.75 ± 0.22^b^	13.28 ± 0.14^b^	8.96 ± 0.09^b^	4.89 ± 0.06^b^
	AA	0.165	7.51 ± 0.12^b^	20.03 ± 0.33^b^	12.82 ± 0.22^b^	8.53 ± 0.01^c^	4.61 ± 0.06^c^

1 HL – head length; PecAL – pectoral-anal length; AL – abdominal length; PostAL – post-anal length; HH – head height;

a,b,c –different superscripts within the same row indicate significant difference between haplotype blocks at P-value < 0.01

### *In silico* analysis results

**Fig.3.**
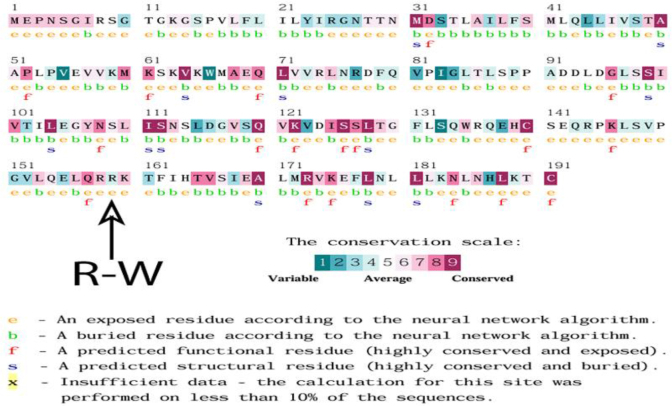
ConSurf database analysis of the leptin isoform X1 protein of *Dicentrarchus labrax*

One SNP (g. 11004767C>T) was identified in the third exon of the *lep* gene, resulting in an amino acid substitution from arginine (R) to tryptophan (W). Structural modelling suggested that this mutation may alter the local protein conformation by replacing a positively charged hydrophilic residue with a bulky hydrophobic aromatic residue. The predicted three-dimensional (3D) structure of the leptin protein, generated using AlphaFold, consisted of five α-helices (α1–α5), forming the typical helical bundle structure characteristic of leptin proteins. The overall predicted structure of the leptin protein is shown in Fig. 3. The substitution site was located in close proximity to the α4 helix, which is considered to play a role in receptor binding.

**Fig.4.**
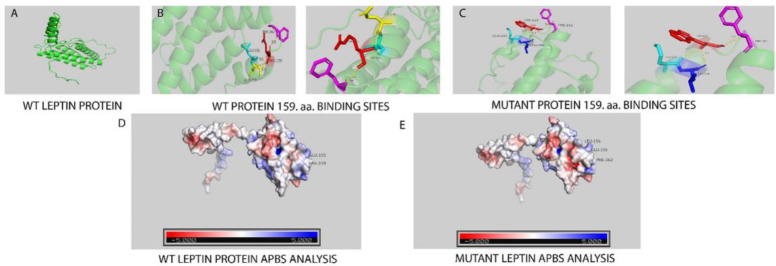
Structural comparison of wild-type and mutant leptin isoform X1. The conservation analysis of the leptin isoform X1 protein predicted that the arginine amino acid located at position 159, which is marked, is not in an evolutionarily conserved region

ColabFold analysis generated five alternative 3D protein structure models. The confidence scores, expressed as predicted local distance difference test (pLDDT) values, ranged from 77.8 to 80.6. Among these models, the third model showed the highest confidence score (pLDDT = 80.6) and was therefore selected for further structural and mutation analyses. The structural comparison between the wild-type and mutant leptin proteins, together with the electrostatic surface potential analysis, is presented in Fig. 4

## Discussion

Growth is a significant trait in aquaculture because of its direct relationship with production efficiency. Its regulation in fish is a complex process controlled by multiple genes and molecular pathways. Candidate gene markers are widely applied in aquaculture genetics because they offer greater specificity and enable the detection of polymorphisms within or near genes that directly influence phenotypic traits (26). Since SNPs associated with growth traits often occur within candidate genes, analysing their relationships with growth performance is an effective strategy for identifying functional variants conferring quantitative traits (39). In Nile tilapia (*Oreochromis niloticus*), polymorphisms in the growth hormone gene have been significantly associated with growth-related traits, highlighting the functional relevance of candidate gene polymorphisms in growth regulation (1). In this context, the *lep* gene is another promising candidate for a gene involved in growth regulation and metabolic control in *D. labrax*. In this study, significant associations between *lep* SNP variants and growth traits were identified in this species.

The discovered SNPs associate with phenotypic variation in this species. Optimally efficient selective breeding relies on genotype identification rather than phenotype selection; the association of genotype with phenotype can be considered reliable when the markers are known to be informative. Polymorphic information content indicates the informativeness and degree of polymorphism of a marker (8). In the present study, four markers showed moderate polymorphism based on their PIC values (0.25 < PIC < 0.5), suggesting that they can be used in genetic diversity studies (34). The deviation of all analysed SNPs significantly from HWE may reflect the breeding history of this cultured stock, in which founder effects and genetic drift may have been manifest (7). Overall, the observed levels of polymorphism and allele frequencies indicated a moderate degree of genetic variation, supporting the applicability of these markers in genetic analyses and marker-assisted selection programmes. Linkage disequilibrium analysis of the five SNPs in the European sea bass population indicated overall low to moderate LD. A relatively strong association was detected between g.11004714G>A and g.11004767C>T (r^2^ = 0.68), which supports the presence of a haplotype structure in this region. Two haplotype blocks were identified, and although LD within the second block (g.11005304A>G and g.11005335G>A) was weaker, the overall pattern provided useful information on the genetic architecture of the population. These results suggest that the inclusion of additional markers could further improve the resolution of association analyses and facilitate identification of growth-related loci.

A triallelic SNP (g.11005020T>C>G) was identified in the first intron of the *D. labrax lep* gene. Although triallelic SNPs are expected to be much less common than biallelic SNPs, their discovery is becoming more frequent because of the growing number of genomic studies on organisms (30). For instance, in humans, 1,270 polymorphic triallelic SNPs have been identified in the 1,000 Genomes Project (28). Such markers have recently been applied in genetic monitoring studies of aquatic species, including salmon (30). Given their reflection of more allelic diversity, triallelic SNPs can be informative markers in various contexts, such as evolutionary studies, individual identification and genetic characterisation of hybridising species (19). They may also serve as valuable markers for population genetics or parentage analysis within a singlespecies such as the European sea bass. Triallelic SNPs can be particularly informative within a single-species context because they capture greater allelic diversity, which is especially useful in cultured *D. labrax* populations where Atlantic and Mediterranean broodstocks are mixed, allowing detection of subtle population structure and hybrid ancestry that biallelic markers may miss.

The results indicated that both synonymous loci g.11004714G>A and g.11004804G>A, located in the third exon of the *lep* gene, were significantly associated with growth-related traits in *D. labrax*. Specifically, the g.11004714G>A locus was associated with TW, FW, SL, BL, PostAL and AL, while the g.11004804G>A locus was associated with PostAL. Both synonymous and non-synonymous exonic SNPs have been recognised as valuable for genetic studies because even synonymous SNPs can influence phenotypic variation (14). Synonymous mutations, traditionally considered neutral, can significantly influence genetic processes by modulating gene expression, thereby producing phenotypic and fitness effects that have important implications for genetics, evolution, conservation and disease research (42). Moreover, synonymous mutations have been reported to affect alternative splicing, splicing efficiency and mRNA stability or turnover (7). For example, Prihandini *et al*. (29) reported that a synonymous SNP (g. 12238G>A) located in the second exon of *lep* was significantly associated with body length in Rambon cattle. Similar findings were reported in six SNPs in the *leptin-a* gene associated with several growth traits of orange-spotted grouper (*Epinephelus coioides*) with two SNPs were located in the first intron and four in the second exon (38). Additionally, Huang *et al*. (15) identified six SNPs in the *leptin-b* gene of the orange-spotted grouper and stated one SNP (c. 149G>A) in the first exon to be strongly associated with growth traits and the heterozygous mutation genotype GA to have negative effects on growth traits. Wu *et al*. (40) reported that the polymorphisms T775A in exon 2 and G1239A in exon 3 of the leptin-a gene were associated with body weight and body length in golden pompano (*Trachinotus blochii*). Individuals carrying the GA genotype G1239A showed superior growth performance. Tang *et al*. (33) conducted association analysis and revealed three SNPs which were significantly associated with growth traits in male Jian carp (*Cyprinus carpio* var. Jian), but only one SNP which was significantly associated with growth traits in females. These results demonstrate that polymorphisms in leptin are associated with growth traits and may be used in marker-assisted selection programmes.

Haplotypes represent distinct arrangements of SNP alleles along a chromosome, and such multi-locus analyses can capture more genetic variation than singlemarker analyses and improve the power of association tests (34). Our findings demonstrate that specific haplotype combinations were closely linked to growth variability in the studied population, with the GTA haplotype in block 1 associated with superior growth performance, and the GCG and GTG haplotypes linked to comparatively inferior growth. Likewise, in block 2, the AG haplotype consistently corresponded to enhanced growth-related traits relative to the other variants, suggesting a potential role for these haplotypes in modulating growth performance. The literature has not reported any studies examining the relationship between leptin gene haplotypes and growth traits in European sea bass, although a sex-specific association between jlLEP-A2 diplotypes of the *lep* gene and growth traits has been observed in Jian carp (33). Comparable effects of haplotypes have been documented in other aquaculture species, and similar findings have been reported for candidate genes. Duan *et al*. (7) found in the swimming crab (*Portunus trituberculatus*) that two of the four SNPs of the *SIF* gene formed a block containing three haplotypes (GC, TA and TC), among which the TA haplotype was significantly associated with superior growth performance compared to the GC haplotype. As another example, Wei *et al*. (38) performed LD analysis in Nile tilapia and found that specific diplotypes of *Myf5, Myf6* and *Myod2* were strongly associated with superior growth, resulting in BW increases of up to 188%. Similarly, again in the swimming crab, a four-haplotype block of three significant SNPs within the *Myf6* gene was reported, and it exhibited superior growth traits compared to the AAA haplotype (8). These results suggest that haplotype-based approaches can be highly informative in identifying genetic variants linked to economically important traits. Consistently with these studies, our results suggested that the haplotype combinations identified here could serve as promising genetic markers for growth-related selection in aquaculture breeding programmes.

The functional implications of the arginine-to- tryptophan substitution at position 159 in the *Dicentrarchus labrax* leptin isoform X1 protein were assessed based on evolutionary conservation, amino acid properties and the literature (24). ConSurf analysis indicated that this residue was located in a non-conserved region, suggesting that it may be less critical for the overall structural stability, although local conformational changes cannot be excluded. The replacement of a positively charged, polar residue (arginine) with a bulky, non-polar aromatic residue (tryptophan) has the potential to alter local folding or protein-protein interactions, depending on the surrounding context. In humans, arginine-to-tryptophan substitutions such as the R105W mutation were associated with altered leptin activity, reduced serum leptin levels and morbid obesity (24). Although direct extrapolation to teleosts requires caution, these findings raise the possibility that R159W substitution in sea bass may similarly influence leptin function. Leptin expression and plasma levels in *D. labrax* correlate with body fat reserves, paralleling observations in mammals (10). Given that the α4 helix may participate in receptor binding, the R159W mutation could affect leptin function by modifying the local structure, thereby altering signalling capacity. Further structural and functional studies are required to clarify its role in energy homeostasis in this species.

## Conclusion

This study initially identified SNPs in the *Dicentrarchus labrax lep* gene and examined their association with growth traits. This gene is recognised for its association with several economically significant traits in livestock, rendering it a promising candidate gene to use to production performance enhancement ends. However, genetic characterisation of the *lep* gene in aquaculture species remains limited. This study is one of the first investigations exploring the association between its polymorphisms and growth-related traits in European sea bass and establishing a positive correlation. The present study offers novel insights into the role of *lep* in European sea bass by identifying SNPs and haplotypes that are potentially linked to growth-related traits. These findings provide a valuable foundation for interpreting the functional significance of *lep* polymorphisms and their potential as molecular markers for genetic enhancement in cultured fish species. Understanding the genetic factors influencing the growth of aquaculture species is crucial for selective breeding.

## Supplementary Material

Supplementary Material Details
