# Supplementary material for: Novel SNPs in the leptin gene: implications for growth performance in cultured European sea bass (Dicentrarchus labrax)
Source: J Vet Res. 2026 Apr 18;70(2):293–302. doi: 10.2478/jvetres-2026-0021 (PMC13334296; doi:10.2478/jvetres-2026-0021)
Supplement: Supplementary file 1 — Supplementary Material Details [file jvetres-2026-0021_sm.pdf]

Supplementary Table 1

Sample	Bird	Breed	Backyard
O.C.1	Ornamental chicken (*Gallus gallus domesticus*)	Silkie	4
O.C.2		Silkie	
O.C.3		Minohiki	
O.C.4		Totenko	
O.C.5		Chabo	
O.C.6		Ko Shamo	
O.C.7		Ko Shamo	
O.C.8		Onagdori	
O.C.9		Ayam Cemani	2
O.C.10		Ko Shamo	6
D.1	Duck (*Anas platyrhynchos domesticus*)	Polish Pekin	1
D.2		Polish Pekin	
D.3		Polish Pekin	
D.4		Khaki Campbell	2
D.5		Khaki Campbell	
D.6		Polish Pekin	3
